# Genome-wide association studies for Alzheimer’s disease: bigger is not always better

**DOI:** 10.1093/braincomms/fcac125

**Published:** 2022-05-17

**Authors:** Valentina Escott-Price, John Hardy

**Affiliations:** 1 Division of Psychological Medicine and Clinical Neurosciences, Cardiff University, Cardiff, UK; 2 Dementia Research Institute at Cardiff, Cardiff University, Cardiff, UK; 3 UCL Institute of Neurology, Queen Square, London, UK; 4 UCL Dementia Research Institute, UCL, London, UK

**Keywords:** Alzheimer’s disease, genome-wide association study, heritability

## Abstract

As the size of genome-wide association studies increase, the number of associated trait loci identified inevitably increase. One welcomes this if it allows the better delineation of the pathways to disease and increases the accuracy of genetic prediction of disease risk through polygenic risk score analysis. However, there are several problems in the continuing increase in the genome-wide analysis of ‘Alzheimer’s disease’. In this review, we have systematically assessed the history of Alzheimer’s disease genome-wide association studies, including their sample sizes, age and selection/assessment criteria of cases and controls and heritability explained by these disease genome-wide association studies. We observe that nearly all earlier disease genome-wide association studies are now part of all current disease genome-wide association studies. In addition, the latest disease genome-wide association studies include (i) only a small fraction (∼10%) of clinically screened controls, substituting for them population-based samples which are systematically younger than cases, and (ii) around 50% of Alzheimer’s disease cases are in fact ‘proxy dementia cases’. As a consequence, the more genes the field finds, the less the heritability they explain. We highlight potential caveats this situation creates and discuss some of the consequences occurring when translating the newest Alzheimer’s disease genome-wide association study results into basic research and/or clinical practice.

## Teaser

Escott-Price and John Hardy report that large genome-wide association studies are reported as identifying loci for Alzheimer’s disease loci. They suggest that many are incorrectly designated because of misdiagnoses inherent in data collection. After genome-wide association studies of dementia have been performed, significant loci should be tested in neuropathologically confirmed data sets before they are designated as Alzheimer’s disease loci.

## Graphical Abstract

**Graphical abstract:**
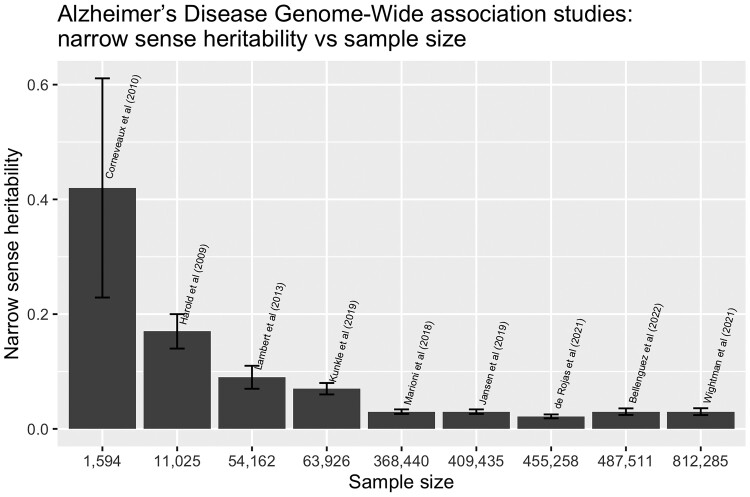

## Introduction

As the size of genome-wide association studies (GWASs) increase, the number of associated trait loci identified inevitably increase.^1^ One welcomes this if it allows the better delineation of the pathways to disease and increases the accuracy of genetic prediction of disease risk through polygenic risk score (PRS) analysis. However, there are several problems in the continuing increase in the genome-wide analysis of ‘Alzheimer’s disease’. The first is that the diagnostic accuracy for Alzheimer’s disease is poor: of the order of 80% in clinic-based series based both on neuropathological criteria^2^ and on genetic analysis^3^ and this is certain to be worse in the case of the proxy cases used in the larger and more recent GWAS. The second is that, while for many rare diseases, age matching of controls makes little difference to the results obtained, because Alzheimer’s disease is a common cause of mortality, the risk gene *APOE* also has the greatest effect on longevity.^4,5^ This makes age-matching essential for accurate risk assessment. In addition, a simple inclusion of age as a covariate in the GWAS creates a robust but biologically spurious association between Alzheimer’s disease and age-associated variants, similar to the association identified between sex- and height-associated variants.^6^ Thus, in case of Alzheimer’s disease, the appropriate use of age-matched controls is important.^7^ A final major problem in the published GWAS is that for most of them, only summary statistics are made available.

These problems are systemic in nearly all the ‘Alzheimer’ GWASs, including ones in which we have been co-authors, except those using neuropathologically defined disease samples^8, 9^ and as data from different studies are meta-analysed together, these systematic errors get baked into the updated analyses. An indicator of diluting the true Alzheimer’s disease associations is the reported heritability estimates. If in a small clinically assessed GWAS (*N* = 11 789 with 3 genome-wide significant loci identified), the heritability was estimated as *h*^2^ = 17% (SE = 3%)^10, 11^: the latest GWAS with a sample size of more than 1.1M people with 38 independent genome-wide significant loci, accounts only for 3% (SE = 0.6%) of heritability.^1^ These errors then get incorporated into PRS analyses and also, perhaps, incorrectly contribute to the suggestion that neurodegenerative diseases share disease mechanisms. In this regard, for example, the designation of *TMEM106B* and *GRN* loci as Alzheimer’s disease loci (both are known frontotemporal dementia loci^12^) is of particular concern, even though they appear in both clinic-based and proxy GWAS data sets. A related problem is likely to be the reported evidence of *APOE* association with clinical frontotemporal dementia (FTD).^13^ What is needed is larger GWAS of Alzheimer’s cases based on either neuropathological or on good biomarker data as, at present, such studies are underpowered. Neuropathological GWAS should give definitive risk loci for disease, whereas GWAS based on biomarker data perhaps give information on disease progression.^14,15^ The danger is that as larger and larger studies of cases with unsatisfactory diagnoses are analysed, the statistical weight behind unwarranted conclusions will become stronger.

## Materials and methods

We have reviewed the GWAS for Alzheimer’s disease derived from analysis of populations of historical European ancestry and assessed their samples sizes, diagnosis and age distributions of cases and controls where possible, as well as the number of genome-wide associated loci they report. The numbers of clinically assessed cases and controls were calculated from the numbers of cases and controls reported in the publication, excluding cases with family-history-based diagnosis (proxy) and controls from the population cohorts in all previous studies contributed to the publication via meta-analysis.

We have extracted the single-nucleotide polymorphisms (SNP)-based heritability estimates for the GWAS from the publications where available and calculated the heritability ourselves if the authors did not provide them in the paper. For the latter, we have downloaded the corresponding summary statistics and used the Linkage Disequilibrium Score (LDSC) regression approach.^16^ We estimated heritability ourselves for fix studies^8,17–21^ using the default settings of the LDSC regression software and pre-calculated LDSCs from the 1000 Genomes European reference population, supplied with the LDSC software. Although Jansen *et al.*^19^ provided heritability estimate for Phase 1 in their Supplemental Note, we have also downloaded the study’s summary statistics, which included the UK Biobank (UKBB; combining Phases 1 and 2). Wightman *et al.*^1^ provided their own heritability estimate, with the same approach, reference population and software options. For the pathology confirmed sample of 1011 cases and 583 controls, we used the summary statistics as reported in Escott-Price *et al.*^9^ Due to the relatively small sample size, the LDSC heritability estimates were negative for these summary statistics when default LDSC parameters were used. Since in the pathology confirmed sample, there were no confounders (such as age mismatching or misdiagnosis), we estimated the heritability for this sample by constraining the intercept using theno-intercept flag.^22^ All heritability estimates were (re)calculated on a liability scale assuming a population prevalence of 5%.

### Data availability

Data sharing is not applicable to this article as no new data were created or analysed in this study.

## Results

**Figure 1:**
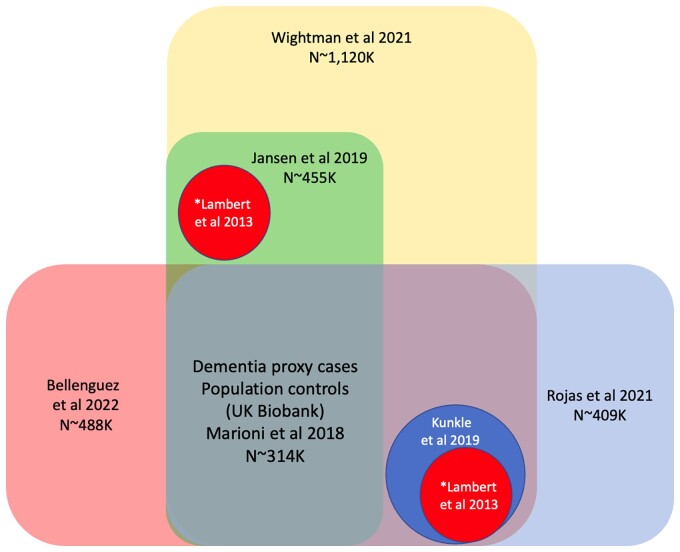
**Overlap of the AD GWAS.** *Lambert *et al*. (2013) and Kunkle *et al*. (2019) are included to Wightman *et al*. (2021) only once.

Apart from four early GWASs (2009–2011), none of the current GWASs are independent (see Fig. 1 and Table 1). The latest GWASs (2019 onwards) include a large proportion of ‘cases’ are based upon the reported impression of offspring that their parent had dementia (usually referred to as ‘proxy Alzheimer’s disease cases’). The accuracy of these impressions is suspect, but, even assuming that 80% of parents have dementia, only 60% of them are likely to have had Alzheimer’s disease. This will introduce significant noise into the data set resulting in about 50% of parental cases having a different form of dementia or no dementia at all. This and any other diagnostic imprecision may specifically limit the detection of variants of small effect, which are the basis of the polygenic architecture of Alzheimer’s disease.

**Table 1:** History of AD GWAS and their SNP-based heritability assuming 5% disease prevalence estimated with LDSC regression^16^

Year	Author	Sample size (Stage 1)			Mean age at assessment^a^		Clinical/pathology assessment (%)		SNP-based heritability on liability scale (5% prevalence)	Number of GWAS significant loci^b^	
		Total	Cases	Controls	Cases	Controls	Cases	Controls		Total	Novel
2010	Corneveaux *et al.*^8^	1594	1011	583	81.9	80.8	100^c^	100^c^	0.42 (0.19)^d^	1	0
2009	Harold *et al.*^10^	11 025	3177	7848	78.6	51	100	26.5	0.17 (0.03)^e^	3	2
2009	Lambert *et al.*^23^	8260	2243	6017	68.5	74	100	100	NA	3	2
2010	Seshadri *et al.*^24^	14 283	1315	12 968	82.7	72.8	100	100	NA	5	2
2011	Naj *et al.*^25^	21 165	10 273	10 892	74.7	76.3	100	100	0.25 (0.02)^e,f^	9	4
2013	Lambert *et al.*^26^	54 162	17 008	37 154	76.6	70.5	100	84.5	0.09 (0.02)^e^	20	11
2019	Kunkle *et al.*^17^	63 926	21 982	41 944	72.9	72.4	100	86.2	0.07 (0.01)	25	5
2018	Marioni *et al.*^18^	368 440	70 306	298 134	Not known	67.3	48.0	18.6	0.03 (0.004)	26	7
2019	Jansen *et al.*^19^	455 258	71 880	383 378	Not known	67.3	33.5	14.4	0.06 (0.01)/0.02 (0.003)^g^	29	13
2021	Rojas *et al.*^20^	409 435	81 611	308 979	Not known	67.3	34.4	13.4^h^	0.03 (0.004)	35	6
2022	Bellenguez *et al.*^21^	487 511	85 934	401 577	67.2	57.9	45.5	14.0	0.03 (0.003)^i^	75	42
2021	Wightman *et al.*^1^	1 126 563	90 338	1 036 225	NA	NA	51.6	9.8	0.03 (0.006)^j^	38	7

^a^ Mean age at assessment (if not reported) was estimated as weighted (by the sample sizes) average of the ages at assessments reported in the contributing studies.

^b^ Using meta-analysis of Stages 1 and 2 (replication) data.

^c^ Pathology confirmed.

^d^ Heritability is estimated using summary statistics of imputed GWAS.^9^

^e^ Transformation to liability scale with 5% prevalence is reported by Zhang *et al*.^11^

^f^ Estimated with GCTA software.^27^

^g^ Without/with UK Biobank data.

^h^ Reported in Moreno-Grau *et al*.^28^

^i^ With UK Biobank data.

^j^ Without UK Biobank data.

The number of clinically assessed controls drops down to ∼10% as the majority of them are population based, and consequently not age matched. If in the pathology assessed GWAS^8^ and (mostly) clinically assessed GWAS,^26^ the average age difference was about 1 year, in the latest GWAS, it is about 10 years or simply impossible to trace (Table 1).

**Figure 2:**
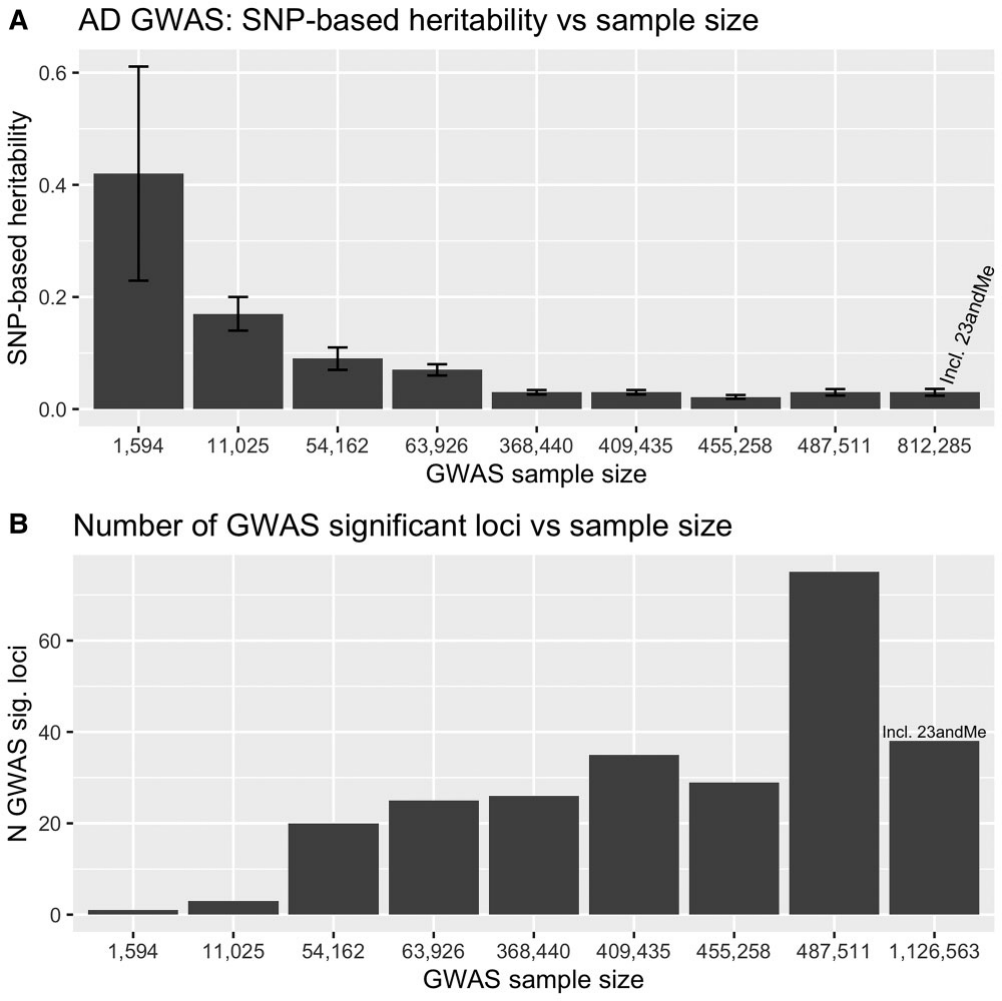
**Relationship between the GWAS sample size and the genetic findings**. (**A**) Heritability. (**B**) The number of novel loci. For Wightman *et al.* (2021) and Bellenguez *et al.* (2022) studies, the heritability was estimated using summary statistics, excluding UK Biobank data.

Counterintuitively, the exponential increase in sample size provides only marginal increases in the identification of novel GWAS significant loci: 2 in the samples of ∼10 000 people,^10,23^ and 7 in the sample of ∼1 126 563 people.^1^ Remarkably, the heritability estimates drop from ∼40^8,9^ to 2–3%^1,18–21^ (see Table 1, Fig. 2) as the sample size increases, despite the fact that all earlier GWAS are included to the latest ones (see Fig. 1).

## Discussion

### Why the heritability estimates are not accurate?

In the context of Alzheimer’s disease, heritability itself is a complex concept since it is possible that everyone would develop Alzheimer’s disease if they lived long enough (but see Morris^29^); and genetic risk appears to determine when this occurs, not if it will occur^30,31^: thus heritability estimates are exquisitely age dependent. Twin studies report heritability of Alzheimer’s disease 59–78%^32^ usually referred as broad-sense heritability. The SNP-based (narrow-sense) heritability estimates are varied across different data sets between 3^1^ and 31%.^33–35^

Different approaches are used for heritability estimates [genome-wide complex trait analysis (GCTA)^27^ and LDSC^16^] with the latter gaining more popularity as it requires only summary statistics. However, the two approaches disagree in their estimates even for the same Alzheimer’s disease data sets, while for neurodevelopmental disorders, the heritability estimates are consistent.^33^ For example, in the same data set,^10^ the estimate is 31% with GCTA and 17% with LDSC.^11^ As LDSC uses only summary statistics, it will not pick up the relatedness between the study participants, specific to neurodegenerative disorders. In particular, there could be a different genetic architecture of *APOE*-ɛ4 carriers when compared with non-carriers.^36^ Indeed, it is known that the *APOE*-ɛ4 allele frequency decreases with age,^5,37^ while Alzheimer’s disease prevalence increases with age. In neurodevelopmental disorders (where the methodologies agree), the diagnosis is likely to be more precise since the disorder’s age at onset is early in life.^38^

Other traits such as Parkinson’s disease and major depressive disorder (MDD) have incorporated data sets from both UKBB and 23andMe and have not observed a corresponding decrease in heritability or the discovery of few GWS loci than expected.^39,40^ The reason for Parkinson’s disease is likely due to the clinical diagnosis being more precise, than for Alzheimer’s disease. In addition, Parkinson’s disease has lower prevalence in the population, so the addition of unscreened controls does not add much noise. While in MDD the prevalence it is similar to Alzheimer’s disease, it is an earlier onset disorder. Finally, for both disorders, there is no known genetic factor that modifies the age at onset and the rate of mortality (the latter changes the allele frequencies in an age-dependent way).

### Longevity

Potential bias in estimates of the GWAS effect sizes and significance of a locus (and consequently of the heritability) can be introduced, as SNPs are associated with both Alzheimer’s disease and age. The *APOE* is the prime suspect as it is associated with a shorter lifespan^41^ and with other ‘killers’ in the population such as heart disease and stroke.^42–44^ It has been reported that *APOE*-ɛ4ɛ4 carriers have an age at onset of Alzheimer’s disease of about 16 years earlier than *APOE*-ɛ4 non-carriers, and that the frequency of *APOE*-ɛ4 reduces with age from ∼0.18 in the general population to 0.1 in the age group 85+.^37^ Despite this reduced *APOE*-ɛ4 frequency in the very old (85+), Alzheimer’s disease prevalence is higher in this latter age group.

### Lack of study independence

We argue that Russian-doll-like GWAS, where larger studies include all smaller ones, carrying the imperfections and amplifying them, does not bring clarity in understanding the Alzheimer’s disease genetic architecture. This GWAS set up with only summary statistics available for the researchers (i) does not allow the exploration of further hypothesis in the substudies, e.g. Alzheimer’s disease predictability by the hypothesis-driven-specific (gene-network) PRS, and (ii) masks the understanding of the Alzheimer’s disease heritability estimates.

### Consequences

Nearly all the ‘Alzheimer’ GWAS suffer from all the criticisms we make, in particular, lack of age matching, poor diagnostic accuracy and lack of data transparency.

This is leading to potentially serious issues (for example drug trials targeted at FTD genes in Alzheimer’s disease cases^45^). This problem relates not only to the primary ‘new’ studies, but also the ones in which they are meta-analysed. If earlier GWAS studies have shown that genetics of Alzheimer’s disease and Parkinson’s disease is distinct,^46^ now papers appear discussing genetic overlap between ‘Alzheimer’s disease’ and Parkinson’s disease. However, ‘Alzheimer’s disease’ cohorts certainly include dementia with Lewy body (DLB) cases and overlap between Parkinson’s disease and DLB is well established.^47^ Thus, in many ways, this genetic sloppiness is having consequences both in terms of the loci associated with disease and therefore passed on to cell biologists and for the utility of PRS analyses for clinical prediction of disease. For example, one of the consequences of the reported low SNP-based heritability is the conclusion that late onset Alzheimer’s disease is oligogenic (∼100 genes),^11^ where the authors assumed 9% heritability in their simulation study, whereas earlier publications suggest that the disease is polygenic (thousands of genes).^9, 48^

### What is needed?

The GWASs have clearly made an enormous contribution to our understanding of Alzheimer’s disease, chiefly through the identification of microglial and brain lipid metabolism^49^ as important risk components, and have focussed attention on the way the brain responds to amyloid deposition.^50^ Larger and larger GWASs now display the law of diminishing returns. A clear distinction needs to be introduced between Alzheimer’s disease GWAS and GWAS for dementia to avoid sending the misleading messages to molecular biologists: the latest big GWAS needs to be labelled as dementia GWAS, not Alzheimer’s disease GWAS. In these dementia GWAS, the Russian doll needs to be unpacked so that the summary statistics for each of them can be made available without an application process.

The consensus on the heritability of Alzheimer’s disease captured by the SNPs needs to be reached. If there is extensive missing heritability, as is widely believed, then epistatic interactions are likely candidates for this missing heritability where risks at unlinked loci act multiplicatively rather than additively. The possibility to detect epistatic loci is widely debated (28). However, this possibility is impaired if the case/control definition is inaccurate, and is forever lost if all that is available are summary statistics.

We need to understand more subtle phenotypic variability within the disease and the genetic factors which influence the rate of decline in disease. In this context, more genotyping of deeply phenotyped sample series and of cases with pathological confirmation are needed. In both cases, consents and protocols are required which permit academic access to individual level data to allow *post hoc* informed cleaning of these data. This would be preferable to ever larger GWAS of poorly characterized individuals. In parallel, we certainly need to understand the architecture of disease in non-European populations, and, within the genes we have already found, the identification of variability which would help disease modelling.

One way forward would be to develop a framework where the ever larger dementia GWAS hits were systematically evaluated in GWAS derived solely from Alzheimer’s disease pathologically confirmed samples, independent from the dementia GWAS. The current research trajectory will lead to ever more confusion, especially amongst those who are not aware of the problems we outline.

## Abbreviations

FTD =: frontotemporal dementia
GWAS =: genome-wide association study
LDSC =: linkage disequilibrium score
MDD =: major depressive disorder
PRS =: polygenic risk score.
